# Behavior Evaluation Based on Electroencephalograph and Personality in a Simulated Driving Experiment

**DOI:** 10.3389/fpsyg.2019.01235

**Published:** 2019-06-04

**Authors:** Changhao Ding, Mutian Liu, Yi Wang, Fuwu Yan, Lirong Yan

**Affiliations:** ^1^Hubei Key Laboratory of Advanced Technology for Automotive Components, Wuhan University of Technology, Wuhan, China; ^2^Hubei Collaborative Innovation Center for Automotive Components Technology, Wuhan, China

**Keywords:** personality, electroencephalography, steering behavior, simulated driving, prefrontal cortex, cognitive state

## Abstract

Assessments and predictions of driving behavior are very important to improve traffic safety. We hypothesized that there were some patterns of driving behaviors, and these patterns had some correlation with cognitive states and personalities. To test this hypothesis, an evaluation of driving status, based on electroencephalography (EEG) and steering behavior in a simulated driving experiment, was designed and performed. Unity 3D was utilized to design the simulated driving scene. A photoelectric encoder fixed on the steering wheel and the corresponding data collection, transmission, and storage device was developed by Arduino, to acquire the rotation direction, angle, angular velocity, and angular acceleration of the steering wheel. Biopac MP 150 was utilized to collect the EEG data simultaneously during driving. A total of 23 subjects (mean age 23.6 ± 1.3 years, driving years: 2.4 ± 1.6 years, 21 males and two females) participated in this study. The Fuzzy C-means algorithm (FCMA) was utilized to extract patterns of driving behavior and the cognitive state within the window width of 20 s. The behaviors were divided into five kinds, i.e., negative, normal, alert, stress, and violent behavior, respectively, based on the standard deviation of steering wheel data. The cognitive states were divided into four kinds, i.e., negative, calm, alert, and tension, respectively, based on the EEG data. The correlation of these data, together with the personality traits evaluated using Cattell 16 Personality Factor Questionnaire (16PF) were analyzed using multiclass logistic regression. Results indicated the significance of the cognitive state and seven personality traits [apprehension (O), rule consciousness (G), reasoning (B), emotional stability (C), liveliness (F), vigilance (L), and perfectionism (Q3)] in predicting driving behaviors, and the prediction accuracy was 80.2%. The negative and alert cognitive states were highly correlated with dangerous driving, including negative and violent behaviors. Personality traits complicate the relationship with driving behaviors, which may vary across different types of subjects and traffic accidents.

## Introduction

With the development of the auto industry and an advanced driver assistance system, the accident rates caused by car failure has reduced significantly while human factors play a crucial role. About 80% of collision accidents were related to distraction (CDC, 2014), and in a total of 37,133 deaths on American highways in 2017, more than 35% involved drunk driving or distraction (NHTSA, 2018). Unsafe driving behaviors such as drunkenness, fatigue, and distraction could cause serious accidents and lead to enormous loss of life and property. Effective monitoring of the driver’s status would be very helpful in maintaining the reliability of driving behavior, thereby reducing the occurrence of traffic accidents caused by human error.

Driving is a complex behavior affected by many factors, either long-term (experience, age, disease and disability, alcoholism, drug abuse; self-evaluation of capabilities, driving habit, accident proneness, personality) or short-term (drowsiness, fatigue, acute alcohol intoxication, acute psychological stress, temporary distraction; psychotropic drugs, motor vehicle crime, suicidal behavior, compulsive acts) (Petridou and Moustaki, 2000). The driver’s personality, such as agreeableness, extraversion, and neuroticism, has some correlation with driving accidents (Cellar et al., 2000; Lajunen, 2001; Guo et al., 2016). Drivers with a low score in extraversion, conscientiousness (Guo et al., 2016), and a high score in sensation seeking, driver anger, and normlessness (Brown, 1976) will be more likely related to risky driving behaviors. Young male drivers’ personality traits and tendencies play a major role in predicting risky behavior (Taubman-Ben-Ari et al., 2016).

Fundamentally, driving behavior is controlled by the underlying cognitive process of the human brain. It can be considered as the output of the underlying executive function which regulates thoughts and behaviors including attention, problem solving, decision making, action monitoring, and evaluation (Miller et al., 2016). This cognitive process is affected by many factors, such as consciousness states (attention, alertness, distraction, fatigue) and emotion states (depression, nervousness). Consciousness is the state of awareness of the external or internal object. Attention is the ability to focus and filter relevant stimuli from irrelevant stimuli, and can be selective, divided, or sustained (Miller et al., 2016). Distracted, decreased, or lost attention results in distraction or fatigue. Drivers’ attentional states are very crucial for traffic safety. Previous studies found that drivers with attention deficit hyperactivity disorder such as an impairment in selective attention (Corbett and Stanczak, 1999; Lovejoy et al., 1999; Dinn et al., 2001), divided attention (Tucha et al., 2008), flexibility/set shifting (Hollingsworth et al., 2001; Rohlf et al., 2012), and vigilance/sustained attention (Epstein et al., 2001) may have a higher likelihood to cause or, at least, be involved in traffic accidents. Emotion states such as depression could affect the selective attention of subjects (Joormann and Quinn, 2014) and driving performance such as standard deviation of lateral position of driving (SDLP) (van der Sluiszen et al., 2017). Cognitive processes, which are collective effects on the human brain, of complex factors from external environment and physiological states of drivers, could finally affect normal driving behaviors and stress reactions related to the traffic safety.

Several indexes, such as percent eyelid closure (PERCLOS) (Liu et al., 2008), pupil diameter (Xiong, 2013), or displacement of the driver’s head (Aykent et al., 2014), were utilized to identify cognitive states. Fatigue and high recognition accuracy was mostly obtained. But these indexes could neither directly reflect the mental state nor be applied for direct control of driving behavior. Additionally, fatigue was just one of the factors affecting the cognitive processes that cause traffic accidents and accounted for a small ratio in all traffic accidents, for example, in some countryies like Japan, it accounted only for 1.0–1.5% (Gu, 2009). Prediction of the driver’s cognitive states based on electroencephalography (EEG) signals has been an active area of research in cognitive ergonomics (Sonnleitner et al., 2014; Xiaoling et al., 2016; Hajinoroozi et al., 2017; Lacko et al., 2017). Researchers used EEG to explore the differences of driving behaviors between young and old people and found that older drivers preferred either a rather proactive and alert driving strategy, or a rather reactive strategy (Karthaus et al., 2018). EEG signals contain plentiful information about the underlying cognitive function and can be applied to study the complex information processing procedure (She et al., 2012). Larger 10- to 11-Hz alpha desynchronization at occipital areas was found to relate with compound limb motor imagery task (Yi et al., 2014). EEG has the millisecond-rang temporal resolution, and can objectively and directly reflect the driver’s complicated cognitive function.

During driving, the drivers received a large amount of information. They should adjust their attention, evaluate the behavior of him/herself and the vehicle, balance the risk of traffic accidents and the benefit of driving fast, make decisions, and act accordingly. The frontal gyrus of the human brain plays a crucial role in cognition function including attention (Hsieh et al., 2009), decision-making, executive control, and emotions (Volz et al., 2006), which are all important procedures in driving. The activities of the frontal gyrus will be a good indicator to reveal cognitive states of drivers and, hence, to evaluate the safety of driving behavior.

We hypothesized that there were some patterns of driving behaviors, and these patterns had some correlation with cognitive states and personalities. To test this hypothesis, an evaluation of driving status based on EEG and steering behavior in a simulated driving experiment was designed and performed. Unity 3D was utilized to design the simulated driving scene. A photoelectric encoder fixed on the steering wheel and the corresponding data collection, transmission, and storage device were developed by Arduino to acquire the rotation direction, angle, angular velocity, and angular acceleration of the steering wheel. Biopac MP 150 (Biopac, United States) was utilized to collect the EEG data simultaneously during driving. A total of 23 subjects participated in this study. Their personality traits, evaluated using Cattell 16 Personality Factor Questionnaire (16PF), together with the EEG data near the frontal area, and the steering wheel data were analyzed by using fuzzy C-means algorithm (FCMA) and multiclass logistic regression. Results indicated the significance of cognitive state and seven personality traits in predicting the driving behaviors, and the prediction accuracy was 80.2%. Our work might be helpful for driving behavior prediction and precaution by using EEG and personality traits.

## Materials and Methods

### Method Overview

**FIGURE 1:**
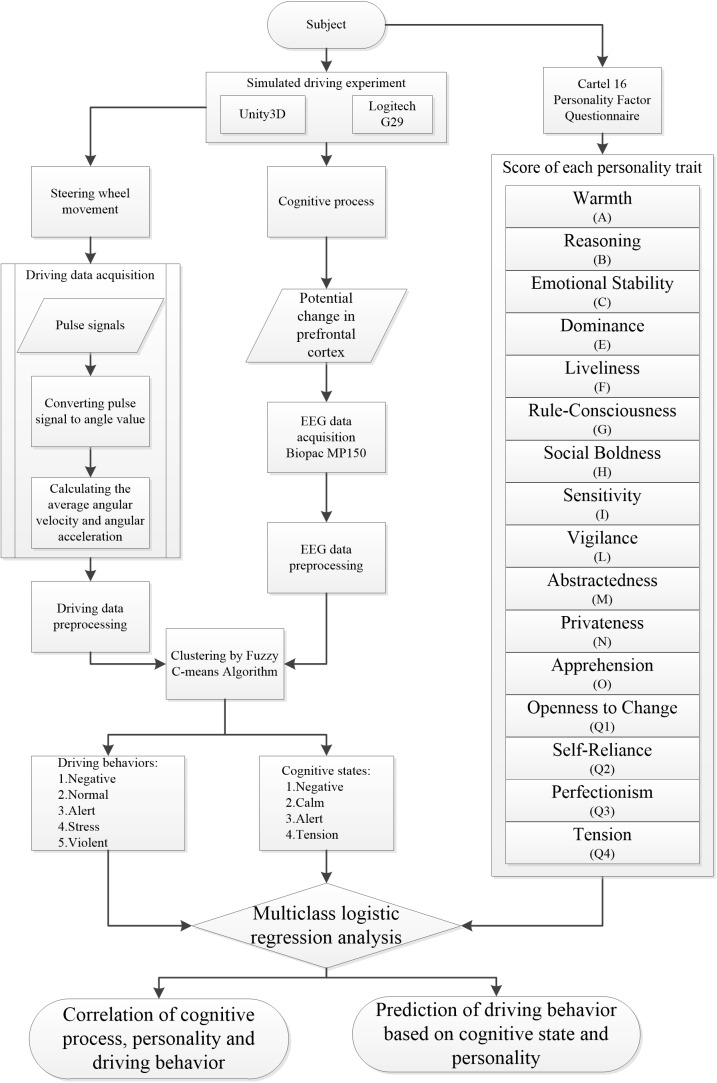
Research flow chart.

The workflow of the whole study is shown in Figure 1. The following steps were included: (i) simulated driving environment design; (ii) driving data, EEG data acquisition, and personality evaluation; (iii) clustering by FCMA; and (iv) multiclass logistic regression analysis.

### Experiment Design

#### Simulated Driving System Designed by Using Unity 3D

**FIGURE 2:**
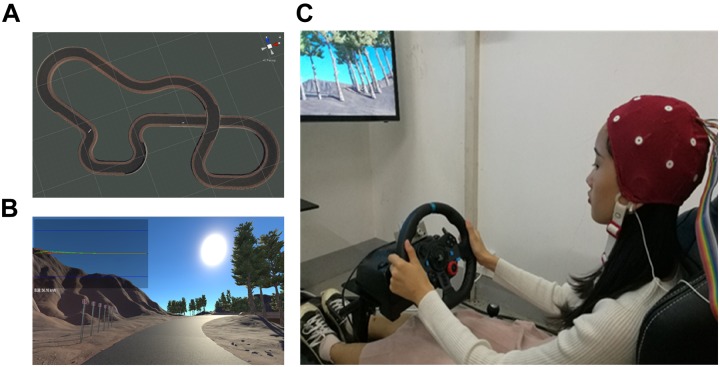
Simulated driving system. **(A)** Driving track, **(B)** driving scenario, and **(C)** simulated driving platform. The subject has provided written consent for the publication of this image.

We established a simulated driving system using Unity 3D (Unity Technologies, Denmark) and Logitech G29 (Logitech, Switzerland). A circular track with total length about 8.5 km was designed containing two consecutive S-shaped curves, two large curved roads with a radius of 20 m, and seven other curves (Figure 2A). The models such as road sign, rock, or vehicle from opposite lane in the resource library of Unity 3D were utilized to simulate the reality world and signs of turning direction before each curve was set to inform the drivers to prepare for the coming turning (Figure 2B). Logitech G29 simulator is the controller of the simulated driving system with force feedback steering wheel, brakes, and clutch.

#### Driving Task

Each driving task contained four rounds of the track. The subjects were instructed to keep their attention on driving and completed two or three driving tasks with a speed limit of 60 km/h. Before the experiment, the subject had enough time (at least 20 min) to get familiar with the acceleration torque of the car, the sensitivity of the steering wheel and the seat, in preparation for the experiment. After each task, the subjects would rest for at least 5 min. The total driving time for every subject was above 30 min. The errors that the driver made during the experiment, including driving out of the lane, colliding with obstacles in the opposite lane, and losing control of the vehicle, were recorded. Subjects with the lowest accident rates would receive extra rewards including a free haircut coupon and a free and expensive meal. We introduced this incentive mechanism to make sure that the subjects would drive as seriously as in their normal states.

A total of 23 subjects (mean age 23.6 ± 1.3 years, driving years: 2.4 ± 1.6 years, 21 males and two females) were recruited in this study. All subjects had driving licenses and reported no neurological or psychiatric problems. All subjects provided prior written informed consent. The study was approved by the ethical review committee of Wuhan University of Technology.

### Data Acquisition

A driving data acquisition device was developed using Arduino Mega 2560 and a photoelectric encoder, which was fastened tightly to the steering wheel using a synchronous belt. The movement of the steering wheel would trigger the rotation of the encoder simultaneously, and the signal would be transmitted to the computer by the serial port at a transmission rate of 128,000 Bd. Subjects’ EEG data were collected by MP 150 with a sampling rate at 1,000 Hz. A total of 16 electrodes covered by Ag/AgCl with a 10–20 system layout (Fz, F8, Cz, Pz, T6, T5, C4, C3, T4, T3, O2, O1, P4, P3, Fp1, and Fp2) were mounted on a recording cap, and one earlobe electrode was taken as the reference electrode (Figure 2C). After the driving experiment, each subject was asked to complete the 16PF Questionnaire.

### Data Processing

#### Definition and Extraction of the Feature Vectors

The rotation angle data were restored using linear interpolation. The transient speed and acceleration were calculated accordingly. Then, these driving data were segmented using 20 s as the window width. The mean and standard deviation of each segment was calculated as feature vectors of driving behavior.

Four channels of EEG data acquired around the frontal area (Fz, F8, Fp1, and Fp2) were first aligned temporally with the behavior data, normalized using the *Z*-score method, and then segmented using 20 s as the window width. The mean and standard deviation of each segment was calculated as EEG feature vectors. MATLAB (R2017a, MathWorks, Natick, United States) was utilized to process the data.

#### Clustering of the Behavioral and EEG Features

Fuzzy C-means algorithm was utilized to cluster the driving feature vectors and EEG features. FCMA uses the fuzzy theory to model the data and divide the data (*n* samples) into *K* clusters (m*_j_* as the cluster center, *j*∈{1,2…*k*}). Each sample *x_i_* is evaluated using *K* membership functions μ*_j_*(*x_i_*), and an objective function embodying the similarity within the same cluster and dissimilarity between different clusters is constructed as follows:

$$J_{f}=\sum_{j=1}^{k}\sum_{i=1}^{n}{\left[\mu_{j}({\text{x}}_{i})\right]}^{b}{\left\|x_{i}-m_{j}\right\|}^{2}$$

where *b* is a weighting exponent on each fuzzy membership and determines the amount of fuzziness of the resulting classification. By optimizing the objective function, an optimal clustering of the data and the membership of each sample was acquired. The number of clusters can be determined by some *a priori* information or using cluster validity procedures such as the “elbow method” (Ketchen and Shook, 1996) or Bayesian information criteria (Neath and Cavanaugh, 2012).

#### Multiclass Stepwise Logistic Regression Analysis

Multiclass forward stepwise logistic regression analysis was performed to determine the correlation between driving behavior and EEG features, by taking the clustering result of the driving features as a dependent variable, the clustering result of EEG features as an independent variable, and scores of the 16PF traits as the covariates. This analysis was performed using SPSS 22.0 (IBM, United States).

## Results

A total of 1,630 samples from 23 subjects were clustered. The driving data were clustered into five categories and EEG data into four categories. Each dimension of the feature vector of the clusters was sorted. The one with the largest value had five votes, and the one with the smallest value had one vote. The total vote of each cluster was obtained by summing these votes together, and the clusters were ordered accordingly. The driving behavior clusters were ordered and termed as “Negative,” “Normal,” “Alert,” “Stress,” and “Violent,” respectively. The EEG clusters were ordered and termed as “Negative,” “Calm,” “Alert,” and “Tension,” respectively. The details listed in Tables 1, 2.

**Table 1:** Original cluster centers of cognitive states.

Clusters	Fz	F8	Fp2	Fp1	Total votes
Tension	0.1080	0.1050	0.1020	0.0998	15
Alert	0.1030	0.0948	0.1040	0.0368	13
Calm	0.0583	0.0557	0.0944	0.0329	8
Negative	0.0213	0.0212	0.0301	0.0201	4

**Table 2:** Original cluster centers of driving behaviors.

Clusters	Angle	Angular speed	Angular acceleration	Total votes
Violent	0.6570	0.40500	0.60	11
Stress	0.0253	0.02170	10.30	10
Alert	0.0562	0.00515	1.49	9
Normal	0.0507	0.00723	1.19	8
Negative	0.0495	0.00432	1.71	7

### Model Fitting Information

The result of multiclass logistic regression analysis is shown in Table 3. The EEG factor and seven personality traits in all 16PF [apprehension (O), rule consciousness (G), reasoning (B), emotional stability (C), liveliness (F), vigilance (L), and perfectionism (Q3)] were significant (*P* < 0.05). The model fitting test indicated −2 times log likelihood of intercept only; the final models were 2,735.193 and 714.291, respectively, and the model was significant (χ^2^ = 2,020.902, *df* = 40, *P* = 0.000).

**Table 3:** Likelihood ratio test results of the regression model.

Effect	Model-fitting criteria	Likelihood ratio test		
	
	−2 log-likelihood value of the simplified model	Chi-square	df	*P*
Intercept	714.291^a^	0.000	0	.
Cognitive state	10,223.281^b^	9,508.991	12	0.000
Apprehension (O)	1,078.625	364.334	4	0.000
Rule consciousness (G)	1,410.471	696.181	4	0.000
Reasoning (B)	1,089.280	374.990	4	0.000
Emotional stability (C)	797.754	83.463	4	0.000
Liveliness (F)	956.240	241.949	4	0.000
Vigilance (L)	867.224^c^	152.933	4	0.000
Perfectionism (Q3)	1,029.613^c^	315.322	4	0.000

*^a^This reduced model is equivalent to the final model because omitting the effect does not increase the degrees of freedom. ^b^The log-likelihood value cannot be further increased after maximum number of step-halving. ^c^There are singularities in the Hessian matrix.*

### Parameter Estimation

**FIGURE 3:**
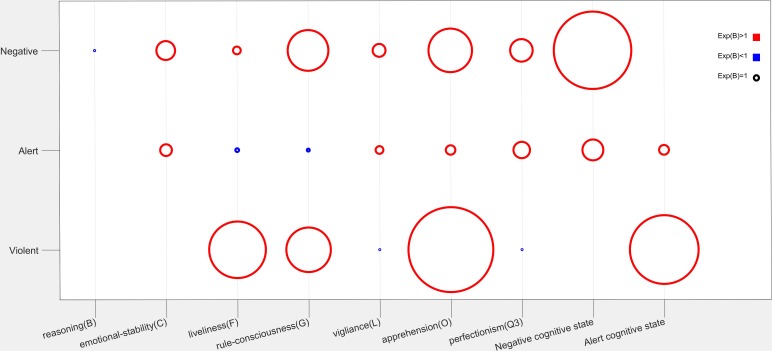
Exp(*B*) (radius of the circle) of the estimated significant parameters in the regression model. The circle with radius equaling 1 was shown in black.

Normal driving behavior and Tension cognitive state in EEG were taken as the reference category. The estimated parameters for Negative, Alert, Stress, and Violent driving behavior using multiclass logistic regression are shown in Figure 3 and Supplementary Table S1.

Negative behavior had a significant correlation with Negative cognitive state [Exp(*B*) = 15.922 *P* = 0.000], apprehension (O) [Exp(*B*) = 8.929, *P* = 0.000], rule consciousness (G) [Exp(*B*) = 8.389, *P* = 0.000], reasoning (B) [Exp(*B*) = 0.195, *P* = 0.000], emotional stability (C) [Exp(*B*) = 3.855, *P* = 0.000], liveliness (F) [Exp(*B*) = 1.574, *P* = 0.000], vigilance (L) [Exp(*B*) = 2.637, *P* = 0.000], and perfectionism (Q3) [Exp(*B*) = 4.605, *P* = 0.000]. Alert behavior had a significant correlation with Negative [Exp(*B*) = 0.000, *P* = 4.305] and Alert [Exp(*B*) = 1.996, *P* = 0.024] cognitive state, apprehension (O) [Exp(*B*) = 1.935, *P* = 0.000], rule consciousness (G) [Exp(*B*) = 0.590, *P* = 0.000], emotional stability (C) [Exp(*B*) = 2.424, *P* = 0.000], liveliness (F) [Exp(*B*) = 0.732, *P* = 0.000],vigilance (L) [Exp(*B*) = 1.581, *P* = 0.000], and perfectionism (Q3) [Exp(*B*) = 3.383, *P* = 0.000]. Violent driving behavior had a significant correlation with Alert cognitive state [Exp(*B*) = 14.128, *P* = 0.0232] apprehension (O) [Exp(*B*) = 17.471, *P* = 0.000], rule consciousness (G) [Exp(*B*) = 9.149, *P* = 0.000], liveliness (F) [Exp(*B*) = 11.626, *P* = 0.000], vigilance (L) [Exp(*B*) = 0.176, *P* = 0.000], and perfectionism (Q3) [Exp(*B*) = 0.188, *P* = 0.000]. Stress behavior had no significant correlation with the cognitive states and personality traits.

### Model Prediction

Table 4 shows the predicted results of driving behavior using the regression model. Of 676 samples in the Negative category, 624 were correctly predicted and the correct rate was 92.3%; of 297 samples in the Normal category, 228 were correctly predicted and the correct rate was 76.8%; of 568 samples in the Alert category, 382 were correctly predicted and the correct rate was also 67.3%; of the seven samples in the Stress category, 0 were correctly predicted and the correct rate was 0%; of 82 samples in the Violent category, 74 were correctly predicted and the correct rate was 90.2%. Of all the 1,630 samples in the five categories, 1,308 were correctly predicted and the correct rate was 80.2%.

**Table 4:** Model prediction results.

Observation value	Predictive value					
	Negative	Normal	Alert	Stress	Violent	Percentage correction
Negative	624	1	51	0	0	92.3%
Normal	20	228	46	0	3	76.8%
Alert	113	73	382	0	0	67.3%
Stress	5	0	2	0	0	0.0%
Violent	5	1	2	0	74	90.2%
Total percentage	47.1%	18.6%	29.6%	0.0%	4.7%	80.2%

## Discussion

In this study, we designed steering wheel acquisition equipment with Arduino Mega 2560 and set up the simulated driving experiment environment using the Unity 3D platform and Logitec G29. A total of 23 subjects participated in the study. The steering wheel data and EEG data were acquired simultaneously, and were clustered using the fuzzy C-clustering algorithm. The driving behavior was divided into five kinds of patterns, and EEG data around the frontal area were divided into four kinds of patterns. A multiclass forward stepwise logistic regression analysis was performed to explore the correlation between driving behavior and EEG patterns, as well as personality traits. The likelihood ratio test indicated the significance of the EEG pattern and seven personality traits in the regression model (Table 3). The total prediction accuracy of the regression model was 80.2% (Table 4).

### Correlation Among Driving Behavior, Cognitive State, and Personality

#### Driving Behavior and Cognitive State Classification

Steering wheel movement has a direct effect on automobile behaviors and driving safety. Emergency steering evasion (ESE) is a typical phenomenon in collision avoidance. There were two typical abnormal steering wheel movements with relatively the largest lane deviation during ESE, one with the largest first peak values of the steering angle, fast steering speed, over steering, and large fluctuations of steering wheel angle and the other with low steering speed and insufficient steering angle to avoid collision (Zhao et al., 2018). The mean and standard deviation of the movement data of the steering wheel were demonstrated to be the robust and consistent with characterization (Das et al., 2012). Some researchers used the sudden correction of the steering wheel within a period of time (window width = 60 s) as indicators to measure the degree of driver’s fatigue (Zhang et al., 2010). In our work, we differentiated the steering wheel data based on the standard deviation. The steering wheel data within a 20-s window width, with relatively the highest standard deviation of angle, angular speed, and acceleration was classified as violent driving behavior, which corresponds to the most radical driving or ESE, represented the intensive modulation of the steering wheel, and was closely related with accidents. The cluster with the relatively lowest standard deviation of angle, angular speed, and acceleration was classified as negative driving behavior, which represented the lowest activity of steering wheel, maintained the steering wheel in a specific state for a relatively long time, and revealed insufficient control of the steering wheel. Normal driving behavior represented the normal, smooth, and safe driving behaviors with moderate modulations of the steering wheel. Stress driving behavior represented the behaviors happening before traffic accidents or emergency corrections of the steering wheel when the drivers realized their errors. Alert driving behavior represented vigilant driving behavior when drivers were alert to the potential danger of the environment. The movements of the steering wheel were adjusted more aggressively than in normal conditions, which can show how to improve driving safety or may also become the precursor of stress driving behavior.

A previous study on epileptic seizures found that the standard deviation of EEG signals at different frequency bands of EEG helps to predict ictal brain activity (during a seizure), which differs from normal brain activity, and their model prediction accuracy of epileptic states was 96.7% (Samanwoy et al., 2007). Similarly, we used the standard deviation of a segment of EEG signals near the frontal area as the indicator of the activation degree or efficiency level of the human brain. Through the voting algorithm, the feature vectors of the cluster center were compared; the four EEG categories were sorted according to the overall activation degrees and termed as Negative, Calm, Alert, and Tension, respectively. A negative cognitive state represented decision-making behavior with the lowest self-awareness of the value system (Volz et al., 2006) and was related with the temporary physiological behavior of attention loss caused by fatigue, distraction, or chemical factors like drugs and alcohol (Dinn et al., 2001; Epstein et al., 2001; Rohlf et al., 2012). An alert cognitive state represented the decision-making behavior with the second highest self-awareness of the value system and alertness. Its occurrence was usually accompanied by highly focused attention caused by threatening information or stimuli (Fox et al., 2001, 2002; Ohman et al., 2001). A calm cognitive state represented decision-making with the third highest self-awareness of the value system and the third highest alertness. Its occurrence was usually companied by accustomed behavior like driving in a familiar road which could be completed due to frequent repetition (Volz et al., 2006). A tension cognitive state represented the decision-making with the highest self-awareness of system value and the highest alertness. Its occurrence was usually accompanied with significant mood swings caused by unexpected threats or emergency like oncoming vehicles or lane intrusion (Fox et al., 2001, 2002).

#### The Regression Model of Driving Behavior

Electroencephalography clusters and seven personality traits [apprehension (O), rule consciousness (G), reasoning (B), emotional stability (C), liveliness (F), vigilance (L), and perfectionism (Q3)] were significant factors (Table 3) in the final significant regression model (χ^2^ = 2020.902, *df* = 40, *P* = 0.000). In the 17 initial independent variables, eight were significant, which implied that as a very complicated behavior, driving does get affected by many factors including both cognitive states and different profiles of personalities.

From Table 4, it can be seen that, in all 1,630 samples, negative behavior appeared 676 times and the frequency was 41.4%, normal behavior appeared 297 times (18.2%), alert behavior appeared 382 times (23.4%), stress behavior appeared seven times (0.4%), and violent behavior appeared 84 times (5.1%). If predicting according to the frequency based on the current data, the rates of correct prediction of the driving behaviors would be 41.4, 18.2, 23.4, 0.4, and 5.1%, respectively. Now, by using the multiclass logistic regression analysis, the correct rates for the five kinds of driving behaviors were 92.3, 76.8, 67.3, 0, and 90.2% and increased by 50.9, 58.6, 43.9, −0.4, and 85.1%, respectively. If there is no extra information, the predicted probability for each driving behavior should be 1/5, and the total predicted accuracy is 20%. Instead of using the regression model, the rate of correct prediction of whole samples has been increased by 60.2 to 80.2%. The prediction accuracy for negative and alert behavior was larger than 90%; while for normal and alert it was about 70%. The regression parameters for stress behavior were not significant, and hence, the prediction for stress was low (0%). This meant that the model cannot explain stress behavior well, but it appeared only seven times and did not have much influence on the total prediction accuracy. In general, these results indicated that the regression model can significantly increase the prediction accuracy.

Detecting the patterns of the driving behavior and using the driver’s personality and cognitive state to predict these patterns is the main purpose of this study. The regression model revealed the complicated relationship between behavior, personality, and EEG features, which will be elaborated in the following section.

#### Correlation Between Cognitive State and Driving Behavior

The likelihood ratio test indicated that the cognitive state was a significant factor (χ^2^ = 9508.991, *P* = 0.000; Table 3). The estimated regression parameters of cognitive states for each driving behavior listed in Supplementary Table S1 revealed that negative behavior had a significant positive correlation with the negative cognitive state [Exp(*B*) = 15.922, *P* = 0.000]; alert behavior had a significant positive correlation with the negative [Exp(*B*) = 4.305, *P* = 0.000] and alert [Exp(*B*) = 1.996, *P* = 0.024] cognitive states; violent behavior had a significant positive correlation with the alert cognitive state [Exp(*B*) = 14.128, *P* = 0.023].

A negative cognitive state was possibly accompanied by temporary physiological behavior of attention loss caused by fatigue, distraction, or chemical factors like drugs and alcohol, which was potentially related with the lesion or dysfunction of the frontal lobe (Dinn et al., 2001; Epstein et al., 2001; Rohlf et al., 2012). There were many curves with different curvatures in the lane, used in the simulated driving experiments, and the acceleration of the virtual vehicle was different compared to real driving, which made the whole driving task challenging. Drivers needed to be highly focussed, pay full attention to the environment and the vehicle, and frequently modulate their behaviors. Drivers under a negative cognitive status had the lowest cognitive decision-making efficiency. They more easily made mistakes in environment sensing or movement selection and performance. These little mistakes accumulate and may finally cause traffic accidents. An alert cognitive state was related with highly focused attention to threatening information or stimuli (Fox et al., 2001, 2002; Ohman et al., 2001). Drivers under the alert state had high decision-making efficiency, and they more easily to realized and corrected mistakes during driving. A alert cognitive state would also occur when a driver had already been involved in traffic accidents due to the negative emotions such as fear (Ohman et al., 2001) and threat-related stimuli (Fox et al., 2001).

Negative driving behavior always occured when the driver was drowsy or even drunk, when there was the lowest movement or even no movement of the steering wheel at all (Das et al., 2012). Alert, stress, and violent driving behaviors usually occured before traffic accidents or during an emergency correction of the steering wheel when drivers realized their driving errors and the underlying risk of an accident (Zhao et al., 2018). When trying to avoid obstacles or correcting the driving trajectory, different drivers had different strategies. Some had a steady strategy with a relatively small steering wheel angle and smooth angular velocity, whereas some turned the steering wheel sharply with a large angle and an angular velocity. The steady drivers usually had a prediction or a calculation of the best turning trajectory, and the latter changed the trajectory sharply which would potentially increase the driving risks such as slipping or losing control. According to the intensity of the movement, alert behaviors represented the steady steering wheel modulation strategy, violent behaviors represented the sharp modulation strategy, and stress seemed to mediate between them (Zhao et al., 2018).

In terms of the movement intensity alert behavior intermediate between negative and violent behaviors, it is interesting that alert behavior was affected by both the specific cognitive states closely related with negative and violent behaviors, respectively, i.e., a negative and alert cognitive state (Supplementary Table S1). Once a negative cognitive state was detected, both negative and alert behaviors would occur, and the former had a higher odds ratio [Exp(*B*) = 15.922 vs. 4.305]; once an alert cognitive state was detected, both violent and alert behaviors would occur, and the former had higher odds ratio [Exp(*B*) = 14.128 vs. 1.996]. Hence, when negative and alert cognitive states were detected, high attention should be paid to the resultant behavior. If it is alert behavior, the current driving is safe; otherwise, either negative or violent behavior would be closely related with risky driving, and some precaution and prevention measures should be taken to avoid possible accidents.

#### Correlation Between Personalities and Driving Behavior

The likelihood ratio test indicated that seven 16PF personality traits, i.e., apprehension (O), rule consciousness (G), reasoning (B), emotional stability (C), liveliness (F), vigilance (L), and perfectionism (Q3) were significant factors (χ^2^ = 364.334, 696.181, 374.990, 83.463, 241.949, 152.933, 315.322, respectively, all *P* = 0.000, Table 3).

According to the regression parameters in Figure 3 and Supplementary Table S1, negative driving behavior had a positive correlation with these personality traits except for reasoning (B) [Exp(*B*) = 0.195]. Alert driving behavior had a positive correlation with these personality traits except for liveliness (F) [Exp(*B*) = 0.732], rule consciousness (G) [Exp(*B*) = 0.590], and reasoning (B) (*P* = 0.238, not significant). Violent driving behavior had a positive correlation with these personality traits except for vigilance (L) [Exp(*B*) = 0.176], perfectionism (Q3) [Exp(*B*) = 0.788], reasoning (B) (*P* = 0.124, not significant), and emotional stability (C) (*P* = 0.564, not significant). Stress behavior had no significant correlation with the personality traits.

16PF research on the accident drivers and safety drivers indicated that tension (Q4) and perfectionism (Q3) were positively correlated with safe driving, while apprehension (O), openness to change (Q1), self-reliance (Q2), and abstractedness (M) were positively correlated with risky driving (Suhr, 1953; Brown, 1976; Hilakivi et al., 1989; Zhang et al., 2009). Research conducted in China found that drivers with higher scores in self-reliance (Q2), emotional stability (C), warmth (A), dominance (E), liveliness (F), social boldness (H) and lower scores in vigilance (L), and self-reliance(Q2) would be more likely to have a traffic violation than safe drivers (Meng and Lian, 2004).

The highly positive correlation of apprehension (O) with negative and violent behaviors, which were classified as dangerous behaviors, was in accordance with previous research. Though apprehension (O) was also positively related with alert behavior, the odds ratio for alert behavior (Exp(*B*) = 1.935) was much smaller compared with those for negative [Exp(*B*) = 8.929] and violent [Exp(*B*) = 17.471] behaviors. People with a high apprehension (O) score tend to be guilt-prone, worrying, insecure, self-reproaching, and anxious, who were prone to negative emotions such as anxiety and depression and some trivial little things (Brown, 1976). Liveliness (F) and rule consciousness (G) had a positive correlation with negative and violent behaviors, and a negative correlation with alert behavior. These results imply that liveliness (F) and rule consciousness (G) are risk factors for dangerous driving. People with a high liveliness (F) score tend to be highly energetic, carefree, and extraverted but lack restraint and self-control (Conn and Rieke, 1994), which may cause such drivers to ignore traffic regulations and to decrease their alertness and effectiveness in an emergency. And it has been revealed that accident drivers tended to have higher liveliness (F) score (Meng and Lian, 2004). People with high rule-consciousness (G) score tend to be dutiful, staid, and rule-bound. Its positive correlation with dangerous driving behavior seemed unreasonable. Rule consciousness may prevent drivers from drinking or over-speeding, but it may not effectively affect their behavior caused by emergency or emotion fluctuation. The extreme rule consciousness would make people to be compulsive, or become the workaholics or perfectionists (Carter et al., 2016). Under emergency when there was no enough preparation time, these people might act inflexibly or panicky, which may result in negative or violent behavior, respectively. These results also implied that different kinds of traffic events demanded different abilities, such as emotion control, flexibility, self-control, and rule consciousness. Because of the complexity of the personality and driving behavior, there existed some inconsistence in the role of personality traits in driving, such as sensitivity (I), which was the protective factor for safe driving in Brown and Hilakivi’s researches (Brown, 1976; Hilakivi et al., 1989), but the risk factor for dangerous driving in Zhang’s research (Suhr, 1953; Zhang et al., 2009). This inconsistence may relate with the studied subjects and the types of the traffic accidents.

Vigilance (L) and perfectionism (Q3) were positively correlated with negative and alert behaviors, but negatively correlated with violent behavior. People with high vigilance (L) score tend to be suspicious and independent. People with high perfectionism (Q3) score tend to be perfectionistic, self-disciplined, organized, and self-sentimental (Conn and Rieke, 1994). There were no consistent results about their roles in safe or dangerous driving. But it seemed that the drivers with these personality traits can be prevented from modulating the steering wheel too intensively.

### Driving Simulation and Experiment Design

#### Customization of the Simulated Driving Environment

Simulating real driving as similar as possible might ensure the physiological response of the subjects is as normal as during real driving. Driving scenario and automobile operation had the most direct effect on the intuitive feelings of the subjects for the simulated driving experiment. The track model was modified by placing warning signs before every turn, and the number of obstacles such as huge rocks and retrograde vehicles was increased to induce different driving behaviors and the cognitive states of the drivers. Vehicle parameters, such as weight (1.5 t) and suspension vibration frequency (1 Hz), were adjusted according to a normal family car. Maximum torque and real-time torque of the car were set according to the principles of automotive dynamics in real driving. Instead of applying the differential physical model to calculate the angle of wheels based on the real inner and outer wheel angle of the car, the inner steering wheel control program of Unity 3D used the average angle, which made the simulated car more likely to slip and thus increasing the accident risk compared to real driving. Hence, we decreased the maximum angle of the steering wheel to 30° to reduce the occurrence of tire slipping and to improve the operability and comfort of simulated driving. There was no physical feedback from the facilities of the simulation platform, which would greatly affect the feeling and thus the decision-making process of subjects. To address this problem, the slip ratio and vibration of the suspension, as well as the current speed, were displayed on the screen. The high deviation of the slip ratio and vibration of suspension from the baseline, implied the high possibility of losing control. The subjects were instructed to take note of these data and to modulate their behavior accordingly.

#### Incentive Mechanisms

The incentive mechanisms were introduced to encourage a better driving performance. In our experiment, we assumed the difficulty of driving as safely as possible was not much more difficult than driving less carefully. We also offered the driver with the least number of accidents an additional reward (a free and expensive meal at a fine dining restaurant, and a hairdressing coupon) to lure the driver to balance the risk of every behavior during experiments. Like driving license suspension, which is a non-monetary sanction to incapacitate dangerous individuals and deter most drivers from infringing the law (Bourgeon and Picard, 2007), the subjects were told that their driving data would be abandoned if there were too many accidents. Reward-based associative learning had a great effect on driving behavior (Behrens et al., 2008). Both positive effects (highly focused) and negative effects (anxious, ashamed, and angry when making mistakes) were observed in the subjects.

### Data Processing

#### Brain Area Selection

The human brain is a complex organization of information reception, processing, integration, and transmission. Driving is a complicated behavior which should be fulfilled by multiple sensory and cognitive functions of different brain regions. The external information about the environment and the vehicle is censored, decisions are made, and then the corresponding movements of the body are made. During this procedure, several areas should cooperate with each other. Information from the spatial senses converges within the parietal cortex, and is then fed forward to the premotor cortex and integrated with information from the frontal cortex, about action goals and contexts, before the final motor output is sent to the motor areas such as the sensorimotor cortex and primary motor cortex, relayed *via* the corticospinal tracts, and modulated by the cerebellum and basal ganglia (Ball et al., 2008; Gallivan et al., 2013).

The functions of the frontal cortex in cognitive processes has been explored in many studies (Christoff and Gabrieli, 2000). The frontal cortex sub-serves executive control, that is, the ability to select actions or thoughts in relation to internal goals (Koechlin and Summerfield, 2007). During distracted driving, brain activation shifts dramatically from the posterior, visual, and spatial areas to the frontal cortex (Li et al., 2009). Frontal activation is also involved in alerting responses to adapt to challenges in the environment (Richard et al., 2004). As we intended to study the related factors of attention and decision-making in driving, and as the frontal lobe is considered as the control center, we focused on the EEG signal acquired near the frontal lobe (Fz, F8, Fp1, and Fp2).

#### Data Analysis Method

The temporal window width for data analysis was 20 s, and the steering wheel and EEG data within this window were clustered; hence, both the behavior and the cognitive states were described in terms of patterns in a period of time instead of the real-time activities. Some detailed information within this window was filtered. The quantitative effect of the window width on the results, and accuracy of the multiclass regression analysis is worth further researching. Additionally, the application of a moving window on the signal might increase the real-time capability of the schema.

#### Novelty and Limitations

In this study, the driving behavior, neuroimaging data, and the personality data were analyzed in a unified schema, which provided a new viewpoint to monitor the driving behavior and predict the dangerous behaviors based on the cognitive states and personality traits of the subjects. Most driving safety research utilized self-report tools (Schultheis et al., 2002; Arnedt et al., 2005; Kass et al., 2010) to evaluate subjects’ physiological and psychological states like drowsiness, drunkenness, or distraction, which may possibly induce negative observer-expectancy (Sackett, 1979) and subject-expectancy (Clifford and Maisto, 2000) effects. We utilized the relatively objective indicators extracted from EEG data to depict the different cognitive states, and from the movement of the steering wheel to depict the different behaviors of the subjects. The application of these indicators can avoid the subjectivity of those performing the experiment and the subjects, resulting in more robust and accurate predictions, which are exhibited in our model prediction results (Table 4).

The present study is limited principally by the relatively small sample size, unbalanced gender proportion, and concentrated age of the subject samples. Previous research revealed that age (Aartsen et al., 2002), gender (Rhodes and Pivik, 2011), and education background (Salthouse, 2009) were significant factors affecting human’s cognitive functions and cognitive abilities like inductive reasoning, spatial visualization, episodic memory, and perceptual speed. Our results need to be replicated in a much larger sample size and general population. In this study, the related factors of attention and decision-making in driving was the primary focus, and hence, the EEG signal acquired near the frontal lobe (Fz, F8, Fp1, and Fp2) was analyzed. Including more areas with sensory and motor functions in the analysis might help to further our understanding of driving behavior. We chose the mean and standard deviation of behavioral and EEG segments as the feature vector, which reflected the characteristics of the dataset in the time domain. Other features in the frequency domain may also contain important information of human cognitive states (Elif et al., 2006; Kisley and Cornwell, 2006; Kanayama et al., 2010). Finding the optimal feature vectors based on multiple characteristics of the dataset might be helpful to optimize the prediction model. Additionally, the method to cluster driving behaviors and cognitive states was FCMA, which is susceptible to the local extremum. Using the fuzzy neural network algorithm by imitating the brain functions such as learning, association, identification, and information processing as the prediction model, may help to solve this problem. The long-term goal of this research is to construct a real-time monitoring system of driving safety, which is dependent on an effective and flexible hardware and software platform, including data acquisition devices, real-time data analysis methods, and executive equipment. The CPU clock speed and serial port baud rate of the driving data acquisition device need to be optimized, and the offline clustering and regression methods should be modified and improved in order to supply real-time serial analysis results.

## Conclusion

The EEG and steering wheel movement data was acquired simultaneously in a simulated driving experiment. Based on the EEG data, the cognitive states of the driver were divided into four clusters, i.e., negative, calm, alert, and tension; based on the steering wheel data, the driving behaviors were divided into five clusters, i.e., negative, normal, alert, stress, and violent. The cognitive state and seven personality traits [apprehension (O), rule consciousness (G), reasoning (B), emotional stability (C), liveliness (F), vigilance (L), and perfectionism (Q3)] were significant factors in predicting driving behaviors. The regression model was significant, and the prediction accuracy was 80.2%. Negative and alert cognitive states were highly correlated with dangerous driving, including negative and violent behaviors. Personality traits showed a complicated relationship with driving behaviors, which may vary across different types of subjects and traffic accidents.

## Ethics Statement

This study was carried out in accordance with the recommendations of the ethical review committee of Wuhan University of Technology with written informed consent from all subjects. All subjects gave written informed consent in accordance with the Declaration of Helsinki. The protocol was approved by Wuhan University of Technology.

## Author Contributions

LY and FY was awarded the grant that supported the manuscript. CD designed the data acquisition device and simulated the driving experiment. CD, YW, and ML obtained the data set. CD and LY analyzed the data and wrote the manuscript.

## Conflict of Interest Statement

The authors declare that the research was conducted in the absence of any commercial or financial relationships that could be construed as a potential conflict of interest.
