# Supplementary material for: Behavior Evaluation Based on Electroencephalograph and Personality in a Simulated Driving Experiment
Source: Front Psychol. 2019 Jun 4;10:1235. doi: 10.3389/fpsyg.2019.01235 (PMC6558165; doi:10.3389/fpsyg.2019.01235)
Supplement: Supplementary file 1 [file Table_1.docx]

Table s1. Estimated parameters of the multi-class logistic regression model

| Driving behavior^a^ | Parameters | *B* | Standard Deviation | Wald | *P* | Exp(*B*) | 95% confidence interval for Exp(*B*) | |
| --- | --- | --- | --- | --- | --- | --- | --- | --- |
|  |  |  |  |  |  |  | Lower limit | Upper limit |
| Negative | Intercept | -32.668 | 2.567 | 161.960 | 0.000 | - | - | - |
|  | Negative | 2.768 | 0.362 | 58.495 | 0.000 | 15.922 | 7.834 | 32.362 |
|  | Calm | -0.567 | 0.335 | 2.866 | 0.090 | 0.567 | 0.294 | 1.094 |
|  | Alert | 0.278 | 0.339 | 0.675 | 0.411 | 1.321 | 0.680 | 2.566 |
|  | Tension | 0^b^ | - | - | - | - | - | - |
|  | apprehension(O) | 2.189 | 0.163 | 179.965 | 0.000 | 8.929 | 6.484 | 12.294 |
|  | rule-consciousness(G) | 2.127 | 0.166 | 163.875 | 0.000 | 8.389 | 6.057 | 11.618 |
|  | reasoning(B) | -1.636 | 0.132 | 153.441 | 0.000 | 0.195 | 0.150 | 0.252 |
|  | emotional stability(C) | 1.349 | 0.190 | 50.514 | 0.000 | 3.855 | 2.657 | 5.593 |
|  | liveliness(F) | 0.454 | 0.077 | 34.325 | 0.000 | 1.574 | 1.353 | 1.832 |
|  | vigilance(L) | 0.970 | 0.095 | 104.736 | 0.000 | 2.637 | 2.190 | 3.175 |
|  | perfectionism(Q3) | 1.527 | 0.116 | 174.045 | 0.000 | 4.605 | 3.670 | 5.778 |
| Alert | Intercept | -14.314 | 2.065 | 48.063 | 0.000 | - | - | - |
|  | Negative | 1.460 | 0.327 | 19.948 | 0.000 | 4.305 | 2.269 | 8.168 |
|  | Calm | 0.437 | 0.282 | 2.394 | 0.122 | 1.548 | 0.890 | 2.693 |
|  | Alert | 0.691 | 0.306 | 5.103 | 0.024 | 1.996 | 1.096 | 3.635 |
|  | Tension | 0^b^ | - | - | - | - | - | - |
|  | apprehension(O) | 0.660 | 0.142 | 21.535 | 0.000 | 1.935 | 1.464 | 2.558 |
|  | rule-consciousness(G) | -0.528 | 0.110 | 22.935 | 0.000 | 0.590 | 0.475 | 0.732 |
|  | reasoning(B) | 0.104 | 0.097 | 1.152 | 0.283 | 1.109 | 0.918 | 1.341 |
|  | emotional stability(C) | 0.885 | 0.128 | 47.740 | 0.000 | 2.424 | 1.886 | 3.116 |
|  | liveliness(F) | -0.312 | 0.073 | 18.171 | 0.000 | 0.732 | 0.634 | 0.845 |
|  | vigilance(L) | 0.458 | 0.077 | 35.252 | 0.000 | 1.581 | 1.359 | 1.838 |
|  | perfectionism(Q3) | 1.219 | 0.097 | 157.225 | 0.000 | 3.383 | 2.796 | 4.093 |
| Stress | Intercept | -120.851 | 7689.630 | 0.000 | 0.987 | - | - | - |
|  | Negative | 18.116 | 1522.828 | 0.000 | 0.991 | 73732880.772 | 0.000 | .^c^ |
|  | Calm | 15.846 | 1522.828 | 0.000 | 0.992 | 7620652.974 | 0.000 | .^c^ |
|  | Alert | 15.205 | 2197.674 | 0.000 | 0.994 | 4012133.367 | 0.000 | .^c^ |
|  | Tension | 0^b^ | - | - | - | - | - | . |
|  | apprehension(O) | 4.676 | 452.479 | 0.000 | 0.992 | 107.325 | 0.000 | .^c^ |

Table s1-continued. Estimated parameters of the multi-class logistic regression model

| Driving behavior^a^ | Parameters | *B* | Standard Deviation | Wald | *P* | Exp(*B*) | 95% confidence interval for Exp(*B*) | |
| --- | --- | --- | --- | --- | --- | --- | --- | --- |
|  |  |  |  |  |  |  | Lower limit | Upper limit |
| Stress | rule-consciousness(G) | 0.086 | 570.875 | 0.000 | 1.000 | 1.090 | 0.000 | .^c^ |
|  | reasoning(B) | 0.132 | 0.000 | - | - | 1.141 | 1.141 | 1.141 |
|  | emotional stability(C) | 3.667 | 594.250 | 0.000 | 0.995 | 39.125 | 0.000 | .^c^ |
|  | liveliness(F) | 0.184 | 297.962 | 0.000 | 1.000 | 1.202 | 1.000*(E-13) | .^c^ |
|  | vigilance(L) | 3.656 | 363.934 | 0.000 | 0.992 | 38.722 | 0.000 | .^c^ |
|  | perfectionism(Q3) | 3.153 | 491.271 | 0.000 | 0.995 | 23.415 | 0.000 | .^c^ |
| Violent | Intercept | -24.642 | 7.452 | 10.934 | 0.001 | - | - | - |
|  | Negative | 0.099 | 1.098 | 0.008 | 0.928 | 1.104 | 0.128 | 9.496 |
|  | Calm | 0.345 | 1.091 | 0.100 | 0.752 | 1.411 | 0.166 | 11.974 |
|  | Alert | 2.648 | 1.165 | 5.164 | 0.023 | 14.128 | 1.439 | 138.682 |
|  | Tension | 0^b^ | - | - | - | - | - | - |
|  | apprehension(O) | 2.861 | 0.504 | 32.264 | 0.000 | 17.471 | 6.511 | 46.881 |
|  | rule-consciousness(G) | 2.214 | 0.466 | 22.592 | 0.000 | 9.149 | 3.672 | 22.791 |
|  | reasoning(B) | -0.539 | 0.351 | 2.362 | 0.124 | 0.583 | 0.293 | 1.160 |
|  | emotional stability(C) | -0.266 | 0.461 | 0.333 | 0.564 | 0.767 | 0.311 | 1.891 |
|  | liveliness(F) | 2.453 | 0.348 | 49.569 | 0.000 | 11.626 | 5.873 | 23.017 |
|  | vigilance(L) | -1.738 | 0.394 | 19.477 | 0.000 | 0.176 | 0.081 | 0.381 |
|  | perfectionism(Q3) | -1.671 | 0.446 | 14.026 | 0.000 | 0.188 | 0.078 | 0.451 |

Note. ^a^The reference category is 2. ^b^This parameter is set to 0 because it is redundant. ^c^Floating point overflow occurred while computing this statistic. Its value is therefore set to system missing.
